# Optimizing Molecular Geometries in Strong Magnetic Fields

**DOI:** 10.1021/acs.jctc.0c01297

**Published:** 2021-03-16

**Authors:** Tom J. P. Irons, Grégoire David, Andrew M. Teale

**Affiliations:** † School of Chemistry, University of Nottingham, University Park, Nottingham NG7 2RD, United Kingdom; ‡ Hylleraas Centre for Quantum Molecular Sciences, Department of Chemistry, University of Oslo, P. O. Box 1033 Blindern, N-0315 Oslo, Norway

## Abstract

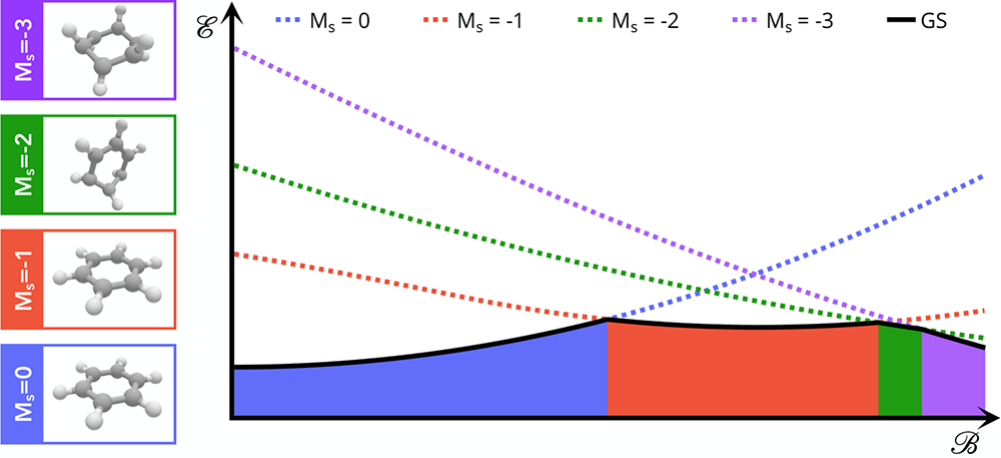

An efficient implementation of geometrical derivatives at the Hartree–Fock (HF) and current-density functional theory (CDFT) levels is presented for the study of molecular structure in strong magnetic fields. The required integral derivatives are constructed using a hybrid McMurchie–Davidson and Rys quadrature approach, which combines the amenability of the former to the evaluation of derivative integrals with the efficiency of the latter for basis sets with high angular momentum. In addition to its application to evaluating derivatives of four-center integrals, this approach is also applied to gradients using the resolution-of-the-identity approximation, enabling efficient optimization of molecular structure for many-electron systems under a strong magnetic field. The CDFT contributions have been implemented for a wide range of density functionals up to and including the meta-GGA level with current-density dependent contributions and (range-separated) hybrids for the first time. Illustrative applications are presented to the OH and benzene molecules, revealing the rich and complex chemistry induced by the presence of an external magnetic field. Challenges for geometry optimization in strong fields are highlighted, along with the requirement for careful analysis of the resulting electronic structure at each stationary point. The importance of correlation effects is examined by comparison of results at the HF and CDFT levels. The present implementation of molecular gradients at the CDFT level provides a cost-effective approach to the study of molecular structure under strong magnetic fields, opening up many new possibilities for the study of chemistry in this regime.

## 1 Introduction

Interest has grown over recent years in the nonperturbative calculation of electronic structure in strong magnetic fields.^1−17^ Such calculations are of interest since they are one means by which static response properties with respect to an applied magnetic field may be evaluated but also since they are essential for modeling the behavior of molecular systems in strong magnetic fields that cannot be treated perturbatively and of the kind found on stellar objects such as magnetic white dwarf stars.^18−20^ Molecular hydrogen has been observed in spectra from nonmagnetic white dwarf stars,^21^ suggesting that molecules and even chemistry may be possible in such extreme environments. The behavior of carbon nanomaterials, such as nanographenes, under strong magnetic fields achievable in laboratories has long been of interest. Nonperturbative response of such systems to a magnetic field is expected as the area of the molecules perpendicular to the magnetic flux increases; for larger systems these effects are predicted to be observable at much lower field strengths.^22^ Nonperturbative effects have also been observed in the context of doped semiconductor materials; see for example the study of Murdin et al.^23^ on phosphorus doped silicon. In recent years, there has been significant progress in extending accurate quantum chemical methods to treat molecular systems under such conditions where standard perturbative treatments are no longer applicable.

Initial developments and investigations concerned the application of Hartree–Fock theory to systems in strong magnetic fields,^1,2^ with subsequent work employing configuration interaction,^24^ coupled-cluster theory,^9^ equation of motion coupled-cluster theory,^10,11^ current–density functional theory,^12,13^ and most recently the calculation of spectra using real-time time-dependent self-consistent field methods.^6−8,17^ Several electronic structure packages have been developed or generalized to treat systems in strong magnetic fields, starting with the London quantum chemistry program^24^ and followed by the Bagel program,^25^ the ChronusQ package,^26^Turbomole,^27^ and our own development code Quest.^28^

Central to these developments has been the use of Gaussian-type London atomic orbitals (LAOs),^29^ which comprise a standard Gaussian basis function multiplied with a complex plane-wave phase factor dependent on the external magnetic field and the gauge origin. Their use allows gauge-origin independent energies and properties of systems in strong magnetic fields to be computed in finite basis sets.

The use of LAOs however has been characterized by computational inefficiency, since the complex phase factor precludes the use of existing optimized molecular integral codes for the evaluation of integrals over LAOs. Furthermore, the algorithms for integral evaluation become more complicated and the operation count itself increases by a factor of 4 when working with complex arithmetic.

In previous work,^15^ an efficient and simple approach for the evaluation of molecular integrals over LAOs was proposed and implemented. A generalized form of the widely employed resolution-of-the-identity (RI) approximation to avoid the need to evaluate four-index electron repulsion integrals (ERIs) over LAOs has been presented and implemented by Reynolds and Shiozaki^30^ and Pausch and Klopper.^27^ Overall, these developments have enabled the computational study of much larger systems in strong magnetic fields than previously possible.

For the study of chemistry in strong magnetic fields, however, it is essential to be able to calculate optimized geometries, and indeed transition states, of molecular systems—since only then is it possible to investigate the changes in molecular structure and orientation relative to the field and ultimately chemical reactivity that occur in strong magnetic fields. Previous work in this area by Tellgren et al. presented analytical gradients in strong magnetic fields for the restricted Hartree–Fock (HF) approach.^3^ A novel approach involving a linear transformation of the basis functions was utilized to generate the required derivatives.

In the present work, a new and simple method is presented for the evaluation of derivative integrals over LAOs, building on the previous approach presented for integrals over LAOs and its implementation.^15^ This leads to a more computationally efficient implementation than the linear transformation method used in ref (3). With these, analytical derivatives of the restricted and unrestricted HF wave functions are constructed and, for the first time, full analytical derivatives of the exchange–correlation (xc) energy in current-density functional theory (CDFT) are presented and implemented. The utility of these methods, and the complexity of chemistry under the influence of a magnetic field, is demonstrated with a detailed analysis of the electronic structure and optimized geometries of two small molecules in strong magnetic fields.

This work is organized as follows: Section 2 contains an overview of LAOs and details the generalization of common intermediates used in integral evaluation to use with LAOs. In Section 3, the evaluation of molecular integrals over LAOs is summarized; this is immediately followed by the extension of these methods to derivative integrals over LAOs, derived in Section 4. The construction of HF analytical gradients from these is described in Section 5 and the extension to CDFT presented in Section 6. The application of these developments to two systems of interest, the OH diatomic molecule and the benzene molecule, is analyzed in Section 7. An overall perspective on this work and directions for future investigation are subsequently given in Section 8.

## 2 Integrals and Derivative Integrals over LAOs

### 2.1 London Atomic Orbitals

A standard unnormalized Cartesian Gaussian-type orbital (GTO) has the general form
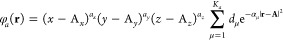
1where the function is centered at **A** = (A_*x*_, A_*y*_, A_*z*_), has angular momentum **a** = (*a*_*x*_, *a*_*y*_, *a*_*z*_), and has *K*_*a*_ exponents {α_μ_} with respective contraction coefficients {*d*_μ_}. LAOs are a generalized form of GTOs,^29^ comprising the form in eq 1 multiplied by a complex phase factor,
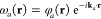
2where **k**_*a*_ is the wave vector of the London plane wave , depending on the external magnetic field  and the position of the LAO relative to the gauge-origin **O**. In the limit of , the LAO in eq 2 will reduce to the corresponding GTO in eq 1. As described in ref (15), the basis may be transformed from the Cartesian representation to the solid harmonic representation with LAOs in the same way as for GTOs, with coefficients constructed explicitly with the method of Schlegel and Frisch^31^ or alternatively by recurrence relation as described in ref (32).

### 2.2 Generalized Shell Pairs

Products of LAOs ω_*a*_(**r**) and ω_*b*_(**r**) represent charge distributions,
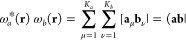
3where the notation [**a**_μ_**b**_ν_| represents the product of the μth and νth individual contractions of ω_*a*_ and ω_*b*_, respectively, while (**ab**| is the overall inner product of the two LAOs; if both are primitive, the two definitions are equivalent.

As is the case for the product of two GTOs, the product of two LAOs yields a further Gaussian function. However, in this generalized Gaussian product theorem, the product Gaussian has its origin in the complex plane; for a pair of primitive *s*-type LAOs, centered on **A** and **B**, with exponents α and β, contraction coefficients *d*_*a*_ and *d*_*b*_, and phase factors **k**_*a*_ and **k**_*b*_, respectively, the product may be written as
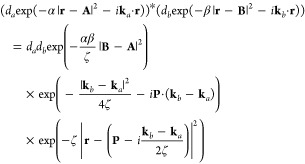
4It follows that the LAO shell-pair for each pair of primitive functions requires only computation and storage of the following quantities
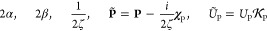
5where
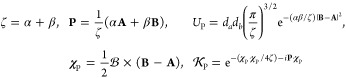
6with which the product of two *s*-type LAOs in eq 4 can be written more concisely as
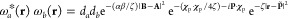
7If |*Ũ*_P_| ≤ 10^–12^, the pair of primitive functions may be considered negligible and discarded from the shell-pair; this allows an increasingly large proportion of the Gaussian product space to be discarded as the system becomes larger. Within this framework of (reduced) shell-pairs, the contraction of eq 3 may be applied as early as possible in each integral algorithm to yield contracted integrals.

### 2.3 London Hermite Gaussian Functions

It is well-established that the product of Cartesian (or spherical) Gaussian functions may be represented as a linear combination of Hermite Gaussian functions;^33−36^ the generalization to London Hermite Gaussian functions can be written as^1,37−40^
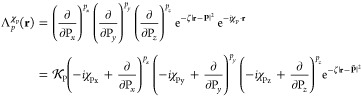
8which may be written, in the notation of eq 3, as [**p**|. The use of Hermite Gaussian functions as intermediates in integral evaluation can provide a computational advantage since, according to the Leibniz theorem, the differential operators over the nuclear coordinates/Gaussian product center can be moved outside of the integral over electronic coordinates. Additionally, higher-order Hermite Gaussian functions can be obtained from lower-order Hermite Gaussian functions by a simple recurrence relation derived as
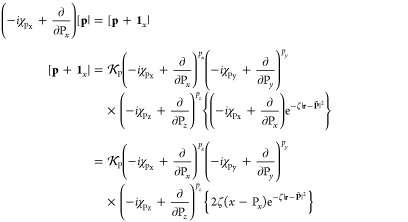
9The term (*x* – P_*x*_) may be moved in front of the differential by using the relation
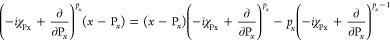
10which may be substituted back into eq 9, allowing the recurrence relation to be obtained as
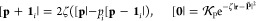
11The transformation between the Hermite Gaussian and Cartesian Gaussian basis can be represented by
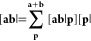
12where the transformation matrices may be obtained by application of recurrence relations, derived as
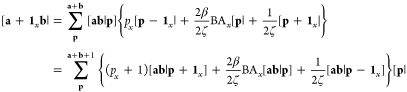
13from which an expression for a transformation matrix element as a linear combination of other transformation matrix elements can be obtained as
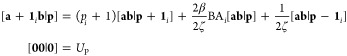
14where additionally [**ab**|**p**] = 0 if **p** < 0 or **p** > **a** + **b**. The transformation between Hermite and Cartesian Gaussian is thus seen to be (i) not explicitly dependent on **P** and (ii) independent of the London phase factor. For convenience in later discussions and consistent with eq 5, *U*_P_ will be included in the Hermite Gaussian prefactor, yielding the modified definitions
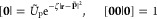
15

## 3 Molecular Integrals

The methods by which derivative integrals are computed are typically generalized forms of the methods for calculating the integrals themselves. In the previous work of ref (15), several approaches for the evaluation of molecular integrals were presented in detail. In the interests of completeness, the aspects of LAO integral evaluation on which the evaluation of derivative integrals depend are reviewed here.

### 3.1 Overlap and Kinetic Energy Integrals

Both the overlap and kinetic energy integrals can be resolved into the product of their Cartesian components, which may be evaluated straightforwardly using the Obara–Saika recurrence relations.^1,15,41,42^ The simplest of these is the two-center overlap integral, defined between two LAOs as
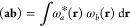
16Using the definition of **P̃** from eq 5, the Obara–Saika recurrence relation for the overlap of two primitive LAOs can be summarized as
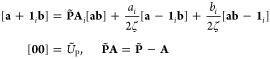
17where the contracted integral is obtained by summation over all primitive contributions and may be transformed into the spherical harmonic basis if necessary.

The kinetic energy integrals can be evaluated as a linear combination of overlap integrals; however, for LAOs their evaluation is complicated by additional terms in the kinetic energy operator, which are not present in the zero-field case. In the Coulomb gauge, the kinetic energy operator is given by half the square of the kinetic-momentum operator , thus the kinetic energy integral is defined as
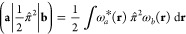
18Denoting the *n*th-order multipole and differential operators respectively as
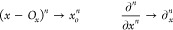
19the *x*-component of the kinetic energy integral may be written as the sum of mixed multipole-moment integrals obtained straightforwardly from overlap integrals described in ref (15),
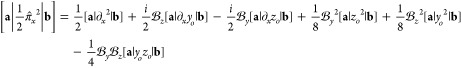
20with which the full kinetic energy integral over primitive LAOs is given by
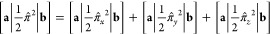
21where contracted integrals are obtained by summation over primitive contributions, followed by transformation to the spherical harmonic basis if required.

### 3.2 Nuclear Attraction Integrals

In contrast to the overlap and kinetic energy integrals, the nuclear attraction integrals (NAIs) are not fully separable into Cartesian components (despite being one-electron integrals) due to the Coulomb operator which defines the integral,
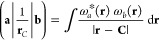
22where **C** is the position of an atomic nucleus with unit charge. In the present work, the Hermite Gaussian expansion described in Section 2.3 will be substituted directly into eq 22, assuming primitive LAOs, as the first step to deriving a method for its evaluation,
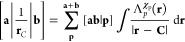
23where the integral may be rewritten using the definition in eq 8 as
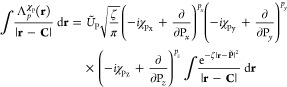
24leaving only an *s*-type Gaussian function, centered in the complex plane, to be integrated over the Coulomb operator. The Coulomb operator may then be eliminated by substituting it with its Laplace transform,
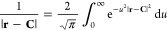
25eventually yielding the one-dimensional integral^43−45^
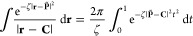
26This one-dimensional integral can be identified as belonging to the class of integrals,
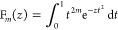
27generally referred to as the *m*th-order molecular incomplete γ function, or the Boys Function.^46^ These functions cannot be evaluated analytically so must be approximated numerically; methods by which this may be done are described elsewhere.^1,27,43,47−49^ For convenience, the following intermediate function is defined, combining the Boys Function with a prefactor by which it is always multiplied
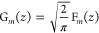
28

Using the result in eq 26, the integral in eq 24 can be simplified to
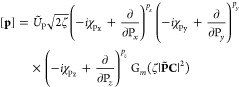
29Hence, integrals over Hermite Gaussian functions are derivatives of the molecular incomplete γ function. Considering the first derivative,
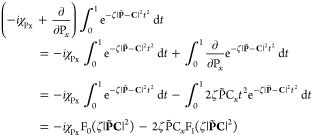
30it can be shown that higher-order derivatives of the incomplete γ function, thus Hermite Gaussian integrals of arbitrary angular momentum, may be obtained from linear combinations of the incomplete γ function at higher order. Defining a class of auxiliary integrals
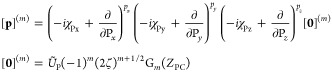
31the order of the Hermite Gaussian integral can be increased as
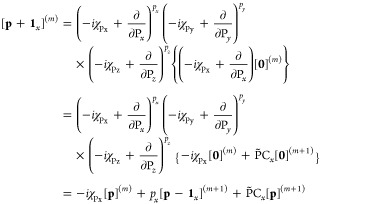
32where the final step involves the use of the relation in eq 10. This yields a recurrence relation for the calculation of all required Hermite integrals from higher-order molecular incomplete γ functions,
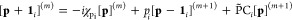
33where, for each primitive integral, the shell-pair data and nuclear position are used to calculate the parameters
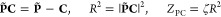
34from which the auxiliary integrals are constructed as
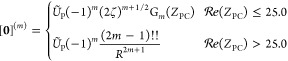
35The integral over the original Cartesian Gaussian functions are obtained by application of the transformation matrix constructed according to eq 14,
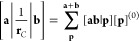
36which may then be contracted according to eq 3 and transformed into the spherical Gaussian basis if required.

### 3.3 Electron Repulsion Integrals

Two-electron repulsion integrals (ERIs) are typically the most computationally expensive to compute, due to the large number which must be evaluated and their individual complexity. In the case of LAOs, the 8-fold permutational symmetry with respect to basis functions for ERIs is reduced to 4-fold, doubling the number of unique integrals to be computed. ERIs over LAOs are defined as
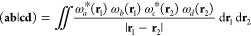
37where the presence of the Coulomb operator again requires a transformation similar to that for NAIs. The derivation of the ERI is very similar to that of the NAI, with a set of shell-quartet parameters^45^ defined by
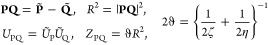
38which may be constructed from shell-pair data, where η, *Ũ*_Q_, and **Q̃** are the second shell-pair equivalents of ζ, *Ũ*_P_, and **P̃**, respectively. To derive the integral expression, it is first necessary to substitute the Hermite Gaussian expansion of a primitive shell-pair into eq 37,
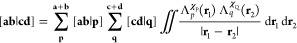
39where the integral may be rewritten making the same re-arrangements as in eq 24, as
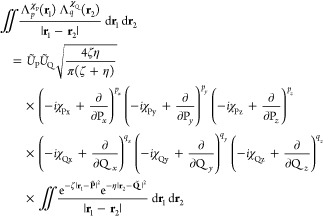
40Using the Laplace transform of the Coulomb operator, this may be reduced to an expression in the incomplete γ function,
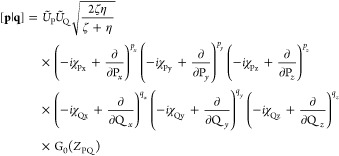
41Recurrence relations are derived analogously to the NAI, defining a class of auxiliary integrals and applying the Leibnitz rule for the *n*th derivative of a product to yield
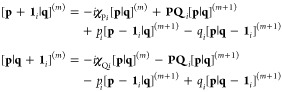
42where the zeroth-order Hermite integrals are evaluated in terms of shell-quartet quantities as
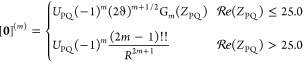
43Finally the integral may be transformed back to the original Cartesian Gaussian basis by application of the transformation matrices in a way similar to that of eq 36 as
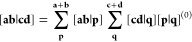
44which can then be contracted according to eq 3 before transformation to the spherical Gaussian basis if required.

## 4 Derivative Integrals

In principle, the calculation of derivative molecular integrals should not be a more complicated endeavor than the calculation of the integrals themselves; since the differentiation is over a nuclear coordinate, it can be moved inside of the integral over electronic coordinates such that the derivative integral can be rewritten as the integral over differentiated Gaussians. Therefore, derivative integrals over Gaussian-type orbitals can be expressed as a linear combination of integrals over Gaussian functions of higher and lower angular momentum.

In practice, the assembly of derivative integrals is somewhat more complex than the integrals themselves since they are much more numerous—each integral is differentiated with respect to the *x*-, *y*-, and *z*-components of each Gaussian center. The complexity increases further when considering the derivatives of LAOs, since differentiation of the phase-factor results in additional terms not otherwise present.

### 4.1 Overlap and Kinetic Energy Integral Derivatives

Following a similar logic to the discussion on integrals, the derivative overlap and kinetic energy integrals are evaluated using the Obara–Saika recurrence relation. Given the product of two primitive LAOs, described in Section 2.2 for *s*-type LAOs and here generalized to higher angular momentum as
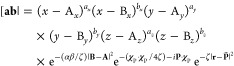
45the derivative of this overlap distribution with respect to the *x*-coordinate of each Gaussian function in turn can be written as
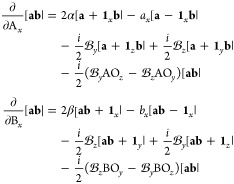
46This translates reasonably straightforwardly into the derivative of the overlap integrals, since this only requires the derivative overlap distribution eq 46 to be integrated over the electronic coordinates. The derivative integral for the overlap of two primitive LAOs is therefore evaluated as
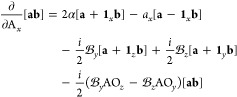
47which can then be contracted and transformed to spherical harmonic representation as appropriate.

The derivative kinetic energy integrals are considerably more complicated to evaluate since the kinetic energy integral over LAOs and in the Coulomb gauge has many more terms than that over standard Gaussian functions. However, the derivative kinetic energy integral remains fundamentally a linear combination of overlap integrals; it is on this basis than the derivative kinetic energy integrals are considered. Starting with the definition,
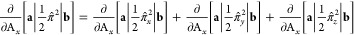
48the derivative of each term must be considered individually, each of which is the sum of several terms shown in eq 20. For each operator required for the kinetic energy integral,
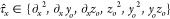
49their contribution to the derivative kinetic energy integral is given by
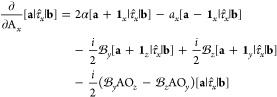
50These terms are assembled into the first term in eq 48, with analogous expressions readily obtained for the π̂_*y*_ and π̂_*z*_ components, to yield the full derivative integral which may be contracted and transformed to spherical harmonics as appropriate.

### 4.2 Nuclear Attraction Integral Derivatives

Derivative NAIs are computed using a method slightly different from that used for derivative overlap and kinetic energy integrals. In Section 3.2, the analytical formulas for calculating the NAIs were presented using Hermite Gaussian intermediates; here it will be shown that this approach can be adapted relatively straightforwardly for the calculation of derivative integrals.

Substituting the differentiated overlap distribution eq 46 into the Hermite Gaussian expansion eq 12 gives an expression for the transformation between Hermite Gaussian functions and the differentiated overlap distribution,
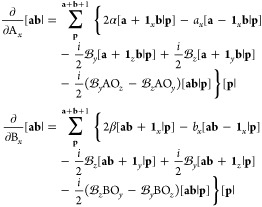
51It follows that a set of differentiated expansion coefficients can be defined as
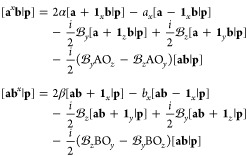
52However, these only account for the derivative with respect to the positions of the two Gaussian basis functions. To compute the derivative with respect to the nuclear coordinate, it is necessary to generalize the definition of the Hermite integral in eq 31 to include a additional index,
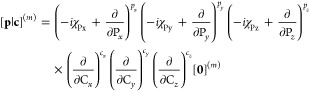
53where the nucleus has no phase factor associated with it. By analogy to eq 42, a set of modified recurrence relations by which the order may be incremented on the nuclear index can be obtained as
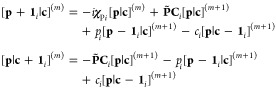
54The derivatives of the nuclear attraction integral may then be assembled as
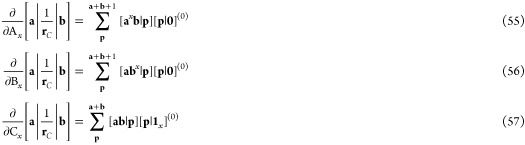
then contracted and transformed into spherical harmonics as necessary.

### 4.3 Electron Repulsion Integral Derivatives

Derivative ERIs are computed in the same way as the derivative NAIs are computed, described in the preceding Section 4.2. Once again, the use of Hermite Gaussian intermediates simplifies the evaluation of integral derivatives since the construction of differentiated expansion coefficients eq 52 enables the derivatives over basis functions to be assembled from the Hermite integrals over an angular momentum range increased by one.
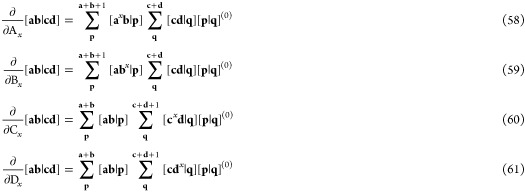


### 4.4 Modified Approach to Constructing LAO Derivative ERIs

The Hermite Gaussian expansion approach in the McMurchie–Davidson algorithm significantly simplifies the construction of derivative integrals.^33,34,50^ However, for LAOs the recurrence relation used to construct the Hermite integrals has significantly more terms than that for standard GTOs and as a result it scales poorly with angular momentum of the shell-quartet.

One approach to improve the efficiency of the derivative ERI computation while retaining the relative simplicity of the McMurchie–Davidson algorithm is by constructing the Hermite integrals directly using the Rys quadrature method.^51−53^ In the Rys quadrature scheme, the zeroth-order terms are not computed from the scaled molecular incomplete γ function but from the standard Gaussian prefactors and the Rys quadrature weights *w*_λ_ as^15,30,53,54^
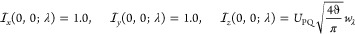
62where the integrand is resolved into the three Cartesian components. For higher-order integrals, angular momentum can be incremented using the following recurrence relations which are analogous to those in eq 42,
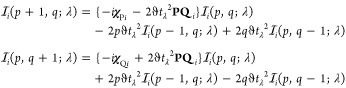
63where *t*_λ_^2^ are the roots of the Rys polynomial. The resolution of the integrand into Cartesian components allows angular momentum to be incremented separately in each direction, resulting in a vertical recurrence relation (VRR) that scales more favorably with angular momentum than that in eq 42 (or indeed the Head-Gordon–Pople recurrence relation,^55^ discussed in ref (15)). Hermite integrals are obtained by multiplying the relevant *x*-, *y*-, and *z*-components of the 2D integrals and summing over the Rys polynomial nodes,
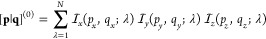
64where *N* is the number of Rys quadrature points; for the case of the integral derivative, *N* = (*L*_*p*_ + *L*_*q*_ + 2)/2. This summation step is generally the computational bottleneck of the Rys quadrature approach, scaling less favorably with angular momentum than the comparatively inexpensive VRR.

To improve the efficiency of this evaluation, Lindh et al. have developed the reduced multiplication scheme for the calculation of integrals^56^ and derivative integrals.^57^ In the construction of [**p**|**q**] integrals, each combination of *x*- and *y*-components is frequently combined with multiple *z*-components; thus, creation of an *xy*-intermediate to be combined with many *z*-components in summation over Rys quadrature nodes can reduce the number of individual multiplications required by the number of quadrature points for each reuse of the intermediate. Additionally, unnecessary multiplication by unity is avoided by discarding  and  from summations where these occur;  cannot be discarded as it carries the Rys weights and other prefactors.

The Hermite integral constructed in eq 64 can simply be substituted into the construction of the derivative ERIs in eqs 58–61 and contracted with the differentiated expansion coefficients to yield the required derivative ERIs. Following the approach of ref (15), in which it was found that the greatest efficiency in integral evaluation may be reached by selecting the appropriate integral algorithm for each shell quartet according to its total angular momentum and contraction length, in the present work the algorithm with which the Hermite integrals were constructed was chosen according to the criteria in Table 1.

**Table 1:** Angular Momentum and Contraction Criteria by Which the Algorithm Used to Compute the Hermite Integrals for a Given Shell Quartet Is Selected^a^

angular momentum	0–2	3–6	7+
primitive	McMD	Rys	Rys
contracted	McMD	McMD	Rys

a McMD refers to the algorithm detailed in Section 3.3, while Rys refers to the method presented in Section 4.4.

## 5 Hartree–Fock Gradients

The force on a given nucleus is evaluated from the contraction of the density matrices with the derivative integrals. Since derivative integrals are computed with respect to the positions of the basis functions in the integral, they do not necessarily contribute to the derivative over each nucleus. For a molecular system, the *x*-derivative of the one- and two-electron integrals with respect to a given nucleus N can be written as^58^
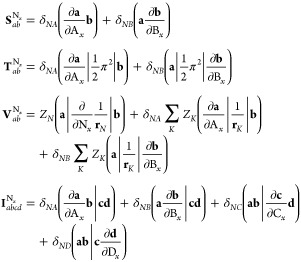
65from which the analytical gradient for a Hartree–Fock wave function is constructed as^3^
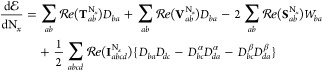
66where *D*_*ab*_ is the density matrix, given by the sum of the spin-density matrices, *D*_*ab*_ = *D*_*ab*_^α^ + *D*_*ab*_^β^, and *W*_*ab*_ is the energy-weighted density matrix, constructed from the spin-density and Fock matrices *F*_*ab*_^σ^ as
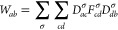
67The contraction of this term with the derivative overlap integrals accounts for the re-orthonormalization of the molecular orbital coefficients in the derivative of the SCF wave function.

### 5.1 Analytical Gradients with the Resolution-of-the-Identity Approximation

The cost of evaluating two-electron integrals can be reduced by employing the RI approximation. In the RI method, two-center charge distributions are expanded in an atom-centered auxiliary basis (**P**,**Q**); four-center integrals are approximated by a contraction of three-center integrals with the inverse of the Coulomb metric of the auxiliary functions
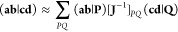
68where the three-center integrals and Coulomb metric are respectively defined as
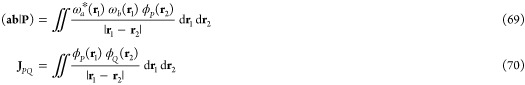
For the auxiliary functions, standard GTOs are used; LAOs cannot be used in the single-center expansion of charge distributions as they would yield an unphysical gauge-origin dependence in the charge distribution. Using standard GTOs for this purpose simply assumes that charge distributions in nonzero magnetic fields are well-approximated by their zero-field equivalents. The use of the RI approximation to accelerate calculations using LAOs is discussed extensively in ref (27).

In the present work, the RI approximation is employed to accelerate the calculation of analytical derivatives over LAOs; derivative electron-repulsion integrals are approximated as
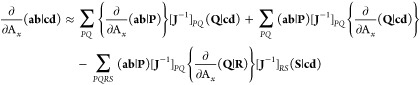
71This construction may be simplified and made more efficient by defining the intermediates,
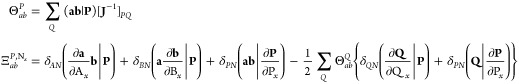
72with which the two-electron derivative integral term is constructed as
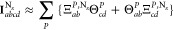
73The judicious use of the Rys quadrature method described in Section 4.4 is also employed in the evaluation of the two-center and three-center derivative integrals used in the RI approximation here, maximizing the advantage that this approach provides.

## 6 Analytical Gradients with Current Density Functional Theory

In the present work, analytical derivatives are also computed for Kohn–Sham current-density functional theory^59−62^ in order to provide insight into the effects of correlation on optimized geometries in strong magnetic fields. In CDFT, the exchange–correlation energy *E*_xc_ is typically approximated at each point in space by some function *f* of local or semi-local quantities
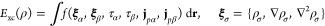
74Functionals which depend only on the density—local density approximations (LDAs), the density, and its first derivative—generalized gradient approximations (GGAs) or the density and its first and second derivatives (some meta-GGAs) are unchanged from their zero-field forms. However, if the functional is dependent on the kinetic energy density τ_σ_ as is the case for most meta-GGAs, dependence on the paramagnetic current density **j**_*pσ*_ is required to ensure the xc energy is invariant with respect to gauge transformation.^63−66^ These quantities, in addition to the electron density ρ_σ_ and its derivatives can be evaluated from the basis of LAOs in which the Kohn–Sham one-electron orbitals are expanded and the spin density matrix as
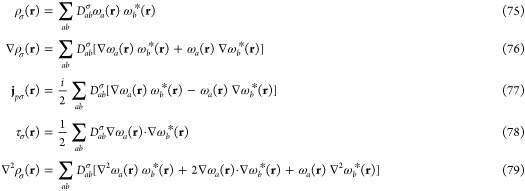


### 6.1 Matrix Elements of the XC Potential

The contribution to the Kohn–Sham potential arising from the xc energy is defined as the functional derivative of the xc energy with respect to the density,
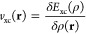
80which, for an xc functional dependent on the density and its derivatives, collectively denoted **ξ** as in eq 74, can be written as^67^
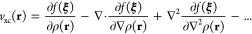
81For meta-GGA functionals with a dependence on the kinetic energy density or indeed the paramagnetic current density, the partial derivative of *f* cannot be written in the same way since these are implicit and not explicit functionals of the density.

In finite basis-set Kohn–Sham calculations, a simplification may be used to evaluate the xc potential contribution to the Hamiltonian matrix,^68^
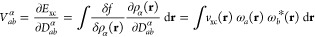
82With the expression in eq 81, this becomes
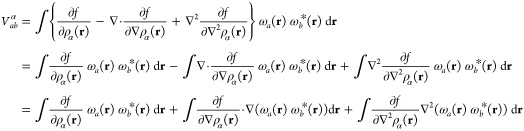
83Since the kinetic energy density and paramagnetic current density are explicit functionals of the density matrix, their contribution to the xc matrix can be computed from the derivative of the energy with respect to the density matrix as an extension of eq 82,
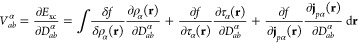
84Combining and simplifying the expressions in eq 83 and eq 82, the total expression for the matrix elements of the xc potential in CDFT can then be summarized as
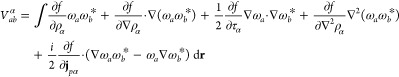
85

### 6.2 XC Contribution to Nuclear Gradient

For a functional of the density and its derivatives **ξ**, the gradient of the xc energy with respect to nuclear position is defined as^67^
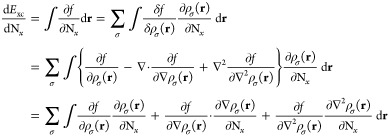
86Derivatives of the kinetic energy density and paramagnetic current density can be evaluated using an approach similar to that of eq 84, yielding an extension to eq 86 of
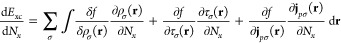
87Collecting the terms from eq 86 and eq 87 together, the xc contribution to the gradient may be evaluated in the AO basis as
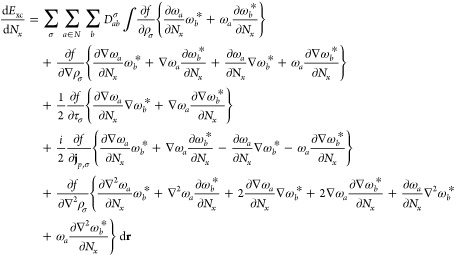
88For completeness, the full expression for the nuclear gradient in Kohn–Sham CDFT is therefore given by
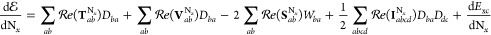
89

## 7 Results and Discussion

The methods for computing one- and two-electron derivative integrals described in Section 4, along with xc analytical derivatives for CDFT described in Section 6.2, have been implemented in the Quest electronic structure code,^28^ which is used in the present work for the geometry optimization of two small molecules in strong magnetic fields. These examples exhibit different, but general, features of chemical structure and bonding in extreme magnetic fields.

Magnetic fields in the order of 0.1B_0_ are known to exist on the surfaces of magnetic white dwarf stars,^18−20^ for which the atmospheres are often dominated by hydrogen and helium but are thought to be abundant in many other elements.^69−71^ Many studies have examined the effects of strong magnetic fields on atomic energy levels,^72−75^ essential for interpreting the spectra observed from these stellar objects. Astrochemical observations also suggest that simple diatomic molecules exist in the atmospheres of magnetic white dwarf stars.^21,76,77^ Modeling the effects of strong magnetic fields on the spectra of molecules has been much more limited than the studies on atoms,^78,79^ although they have become the subject of increasing interest more recently.^5,14^ While molecular studies have become more common, the effects of magnetic field strength on molecular geometry have had much less consideration.^80^ As such, the first system we consider will be the OH diatomic molecule, the properties of which in strong magnetic fields have been the subject of recent astrochemical interest.^81^ In particular, we will see that the behavior of this small system under the strong magnetic fields considered here is well-explained by consideration of orbital paramagnetic interactions with the field.

In ref (82), the potential for conjugated molecules to remain bound even with disruption of their electronic structure was highlighted, noting that even if the π-system is disrupted, the σ-framework could remain intact. In that and more recent studies,^22,82,83^ the Hückel–London approach was used to examine the relative energies of these states as a function of field. In such studies, changes in molecular structure concomitant with changes in the electronic configuration are neglected. As the simplest of the aromatic hydrocarbons, benzene may be considered archetypal for examining the effects of applied magnetic fields on the structure of such systems. As described in ref (22), nonperturbative effects are expected to occur at much lower field strengths for larger conjugated aromatic hydrocarbons such as nanographenes due to their much larger surface area perpendicular to the magnetic flux. In the present work we will see that benzene exhibits significant structural changes in strong magnetic fields. In contrast to the OH molecule, these changes are driven primarily by the spin–Zeeman effect.

### 7.1 Computational Details

In the present work, geometry optimization in the presence of magnetic fields of varying strength is carried out with both HF and CDFT using the cTPSS xc functional,^12,84^ both in the unrestricted formalism. Since both the energy and its first derivative must always be real, a standard quasi-Newton optimization algorithm commonly used in conventional geometry optimizations may be readily employed for optimization in a magnetic field.^85−88^

One important consideration however is the coordinate system that is used; the system of internal coordinates is often convenient in geometry optimization since the coupling between different modes of coordinate displacement, for example representing bond stretching and torsional motion, is minimized.^89,90^ In the presence of a magnetic field, it is not just the positions of atoms relative to each other in the molecule that is important but also the orientation of the molecule with respect to the magnetic field, which is not described by standard internal coordinates.

For all calculations presented in this work a quasi-Newton method, in which the initial Hessian is approximated by the identity matrix and updated at each step with the gradient by the Broyden–Fletcher–Goldfarb–Shanno (BFGS) approach, was employed.^91−94^ Optimization was carried out in Cartesian coordinates,^89,95,96^ in which the orientation of the molecule with respect to the field is most straightforwardly accounted for. In all cases, the convergence criteria were as follows: the largest element of the gradient and of the ensuing step <3 × 10^–4^ au, the root-mean-square of the gradient and of the ensuing step <2 × 10^–4^ au, and the change in energy between steps <5 × 10^–6^ au.

For both molecules considered here, uncontracted forms of the Dunning basis sets^97^ were used to represent the molecular orbitals; uncontracting the basis increases its flexibility and may improve its ability to represent electron densities distorted by application of a strong magnetic field (conditions for which the exponents and contraction coefficients were not optimized).^5,10^ For the OH molecule, the basis used was *u*-aug-cc-pCVTZ while for benzene it was *u*-aug-cc-pVDZ; these two molecules were considered in magnetic fields up to 0.20B_0_ and 0.15B_0_, respectively (B_0_ = ℏ*e*^–1^*a*_0_^–2^ = 2.3505 × 10^5^ T), in which ranges the basis sets selected should provide an adequate description of field-induced density changes.^14,98^

Additionally for benzene, the RI approximation was employed to accelerate the calculations, with the *u*-aug-cc-pVDZ-RI basis used as the auxiliary basis;^99^ recent work has shown that RI may be used with LAOs in a similar way to its use with standard GAOs.^16,27,30^

### 7.2 Equilibrium Geometry of OH

In this section, the equilibrium geometry of the OH diatomic molecule with *M*_*s*_ = −^1^/_2_ in strong magnetic fields is investigated. For this system, the potential energy curve can be correctly represented from equilibrium to dissociation using a single determinant. However, even for this simple molecule, the presence of a magnetic field significantly complicates the potential energy surface, with consideration of the underlying physics required to interpret the optimized geometries obtained.

The nature of chemical bonds can change significantly in the presence of strong magnetic fields, both due to the changes in energy that occur due to the field but also due to the effect of the field on the molecular orbitals themselves. In general terms, a chemical bond may be stabilized or destabilized by the presence of a magnetic field due to the competition between two effects: the change in the energy of the bound molecule with field strength and the change in the energies of the dissociation fragments with field strength. The energy of the bound molecule depends on its orientation with respect to the field, while the energy of the atomic dissociation limits are orientation independent. As the magnetic field strength varies, the energy of the system in these two arrangements may change at different rates and as a result the bonding may be significantly stablilized or destablilzed by the field.^80,100,101^

The presence of the magnetic field can also cause changes in the electronic structure and energy of a chemical bond that cannot be explained in terms of its zero-field electronic structure. In the presence of a magnetic field, the molecular point group may be reduced to one of lower symmetry since only symmetry operations with which the combined molecule and field are unchanged remain. The point group in a magnetic field can be shown to be that comprised of the symmetry operations present in both the zero-field molecular point group and that of a uniform magnetic field: C_∞*h*_. In general, only rotation axes parallel to the field, mirror planes perpendicular to the field, and the center of inversion, if present, will remain.^102^

Therefore, depending on the orientation of the field relative to the molecule, certain symmetries present in the zero-field electronic structure are broken; new types of interactions between molecular orbitals can then result and contribute to the exotic chemistry exhibited in strong magnetic fields. Perhaps the most well-known example is that of perpendicular paramagnetic bonding in H_2_ induced by a strong magnetic field applied perpendicular to the internuclear axis.^103^ A more general analysis of these phenomena has recently been presented by Austad et al. in ref (104).

For the purposes of this work, the effect of the magnetic field on the equilibrium geometry of the OH molecule can be rationalized by considering a magnetic field aligned along the *z*-axis, for which the Hamiltonian can be written as
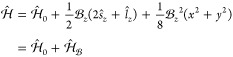
90where  is the zero-field electronic Hamiltonian, *ŝ*_*z*_ the spin angular momentum operator, and *l̂*_*z*_ the orbital angular momentum operator. These terms result in the spin–Zeeman and orbital paramagnetic contributions to the energy in a field, respectively, while the final term yields the diamagnetic contribution to the energy. The spin- and angular momentum-dependent terms can cause an increase or decrease in the energy with respect to field strength, whereas the diamagnetic term will always result in an increase in the energy with field strength; due to its quadratic dependence on , it will always become the dominant term at sufficiently high field strengths.

At zero field, the point group of the OH molecule is C_∞*v*_ and its electronic ground state and first excited state have the electronic configurations
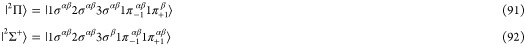
respectively. These dissociate into oxygen and hydrogen atoms in the manner
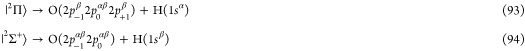
where the different molecular states dissociate into combinations of oxygen and hydrogen atoms of different configurations.^105−107^ For simplicity of notation, the 1*s* and 2*s* orbitals are omitted from the electronic configuration of the oxygen atoms; however, they are occupied as 1*s*^*αβ*^2*s*^*αβ*^ in all cases, and this is assumed throughout.

**Figure 1:**
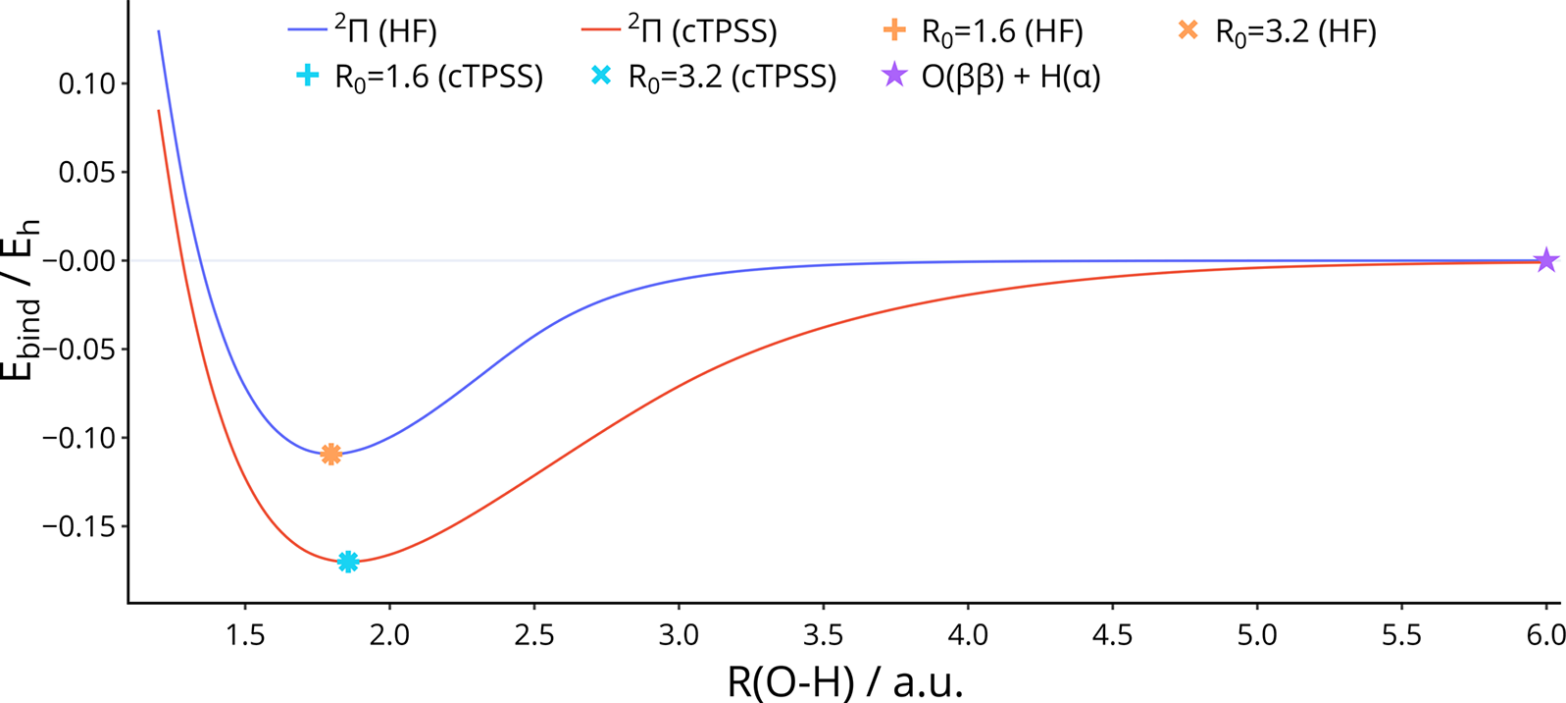
Potential energy curve of OH in the absence of a magnetic field, computed with HF and TPSS. Symbols + and × represent the equilibrium geometries obtained by optimization from initial bond lengths of 1.6 and 3.2 au, respectively. The superposition of both solutions appears as an ∗ symbol. O(2*p*_–1_^β^ 2*p*_0_^*αβ*^ 2*p*_+1_^β^) + H(1*s*^α^) is abbreviated as O(*ββ*) + H(α).

Potential energy curves for the ground state of OH in the absence of a magnetic field, with the optimized geometry and energies relative to the ground-state dissociation products in eq 93 are shown in Figure 1, computed with both HF and TPSS. This confirms that the correct equilibrium geometry of the ground state is located by geometry optimization in the absence of a field using both HF and TPSS, asymptotically approaching the expected dissociation products. The equilibrium geometry of the first excited state would require optimization on an excited-state potential energy surface, not considered in the present work.

At zero field, the ^2^Π state of OH is the lowest in energy for all bond lengths computed, from equilibrium to near-dissociation. Furthermore, the only stationary points along the potential energy curve are at equilibrium and in the dissociation limit; hence, the equilibrium geometry is located easily from different starting geometries. We will consider optimizations starting from 1.6 and 3.2 au here for different field strengths and orientations.

The binding energy at zero field is significantly lower for HF than for TPSS, exhibiting the expected underbinding of diatomic molecules at the HF level as described in, for example, ref (108) at zero-field and ref (103) in strong magnetic fields. Therefore, the present discussion will focus on results obtained with cTPSS; however, the equivalent results obtained with HF may be found in Section S1 of the Supporting Information.

#### 7.2.1 OH in a Magnetic Field Parallel to the Bond

Considering a uniform magnetic field applied parallel to the internuclear axis of the OH molecule, a reduction in symmetry occurs since the ∞σ_*v*_ mirror planes have normals perpendicular to the field and, hence, no longer describe the symmetry of the system in the field. The infinite-order axis of proper rotation along the internuclear axis, *C*_∞_^ϕ^, however remains; thus, the point group of the OH molecule in a field parallel to the internuclear axis becomes C_∞_. The symmetries by which the molecular orbitals in eqs 91 and 92 are characterized remain the same in this case, with the only significant difference being the reduction in symmetry of the excited state from |^2^Σ^+^⟩ → |^2^Σ⟩.

Importantly, since the electrostatic potential and the magnetic vector potential remain cylindrically symmetric around the internuclear axis, the orbital magnetic quantum number remains a good quantum number quantized along the internuclear axis. The initial change in energy of the molecule with respect to magnetic field strength can be approximated from the expectation value of magnetic term of the Hamiltonian eq 90 as
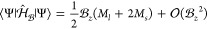
95Thus, the orbital paramagnetic term is proportional to the projection of the orbital angular momentum in the direction of the applied field. The diamagnetic term is proportional to the expectation value of the position squared in the plane perpendicular to the field. It will therefore be nonzero for the molecule in any orientation, but will be minimized when the area of the charge density perpendicular to the field is minimized which, in the case of a diatomic molecule, occurs where the field is parallel to the internuclear axis. Therefore, the expression in eq 95 should provide a suitable model for interpreting the initial behavior of the energy of a diatomic molecule with increasing field strength parallel to the internuclear axis. In this analysis, it is considered that the change in  on application of a magnetic field is small compared to eq 95 over the range of field strengths examined for this system.

For the two electronic states of OH in eqs 91 and 92, their respective initial change in energy with field strength parallel to the internuclear axis can be rationalized with eq 95 as
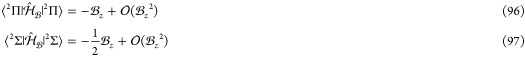
Since both electronic states of OH have *M*_*s*_ = −^1^/_2_, the difference between eq 96 and eq 97 arises due to the difference in the orbital paramagnetic contribution to the energy. It would be therefore expected that the |^2^Π⟩ state would remain the ground state with increasing field strength parallel to the internuclear axis. Geometry optimizations should then be expected to track the change in equilibrium structure of this state as a function of the magnetic field strength.

To investigate this, the ground-state geometry of OH was optimized with magnetic fields of increasing strength between 0.0B_0_ and 0.2B_0_ applied parallel to the internuclear axis. In this orientation, the energy is stationary with respect to the angle between the internuclear axis and the field; thus, without perturbation, the molecule remains in this alignment throughout the optimization. The equilibrium bond length and the respective binding energy (*E*_bind_ = *E*_OH_ – *E*_O_ – *E*_H_) for OH in this series of magnetic fields are summarized in Table 2.

**Table 2:** Equilibrium Bond Length and Binding Energy of OH at Zero Field and with a Magnetic Field Applied Parallel to the Bond Axis, Computed with HF and cTPSS^a^

B/B_0_	*R*_eq_^HF^	*R*_eq_^TPSS^	*E*_bind_^HF^	*E*_bind_^TPSS^
0.00	1.7974	1.8564	–0.10562	–0.17007
0.05	1.7967	1.8546	–0.10982	–0.17288
0.10	1.7954	1.8530	–0.11058	–0.17362
0.15	1.7932	1.8504	–0.11182	–0.17484
0.20	1.7902	1.8469	–0.11348	–0.17645

a Bond lengths are in bohr and binding energies in hartree.

A clear trend can be observed from Table 2 in both the equilibrium bond length and binding energy of OH; the equilibrium bond length decreases with increasing field strength aligned parallel to the internuclear axis, while the binding energy becomes more negative. Both trends have the same interpretation: the molecule is stabilized with respect to increasing field strength parallel to the bond.

**Figure 2:**
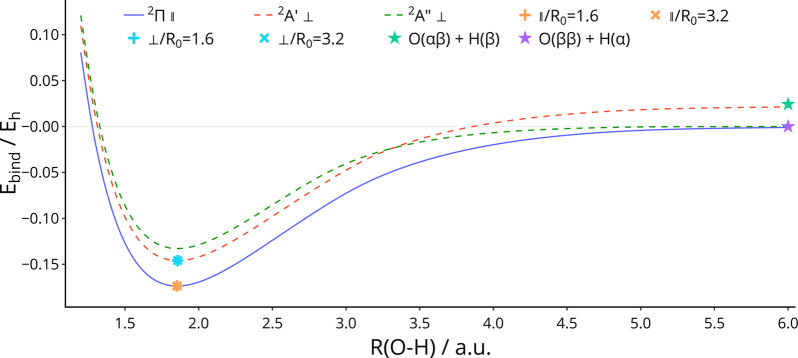
Potential energy curve of OH in a field of 0.10 B_0_ parallel and perpendicular to the O–H axis, computed with the cTPSS functional. Symbols + and × represent the equilibrium geometries obtained by optimization from initial bond lengths of 1.6 and 3.2 au, respectively. The superposition of both solutions appears as an ∗ symbol. O(2*p*_–1_^*αβ*^ 2*p*_0_^β^ 2*p*_+1_^β^) + H(1*s*^α^) and O(2*p*_–1_^*αβ*^ 2*p*_0_^*αβ*^) + H(1*s*^β^) are abbreviated as O(*ββ*) + H(α) and O(*αβ*) + H(β), respectively.

**Figure 3:**
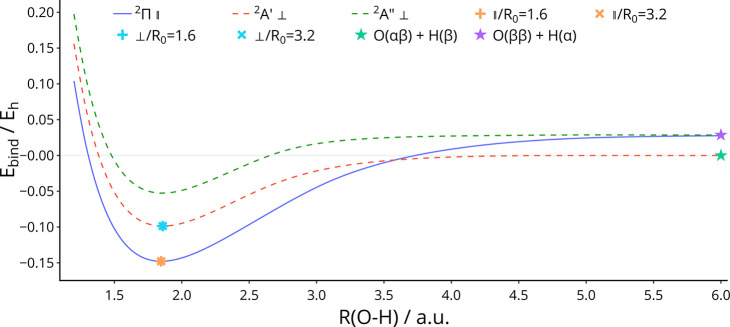
Potential energy curve of OH in a field of 0.20 B_0_ parallel and perpendicular to the O–H axis, computed with the cTPSS functional. Symbols + and × represent the equilibrium geometries obtained by optimization from initial bond lengths of 1.6 and 3.2 au, respectively. The superposition of both solutions appears as an ∗ symbol. O(2*p*_–1_^*αβ*^ 2*p*_0_^β^ 2*p*_+1_^β^) + H(1*s*^α^) and O(2*p*_–1_^*αβ*^ 2*p*_0_^*αβ*^) + H(1*s*^β^) are abbreviated as O(*ββ*) + H(α) and O(*αβ*) + H(β), respectively.

In addition, potential energy curves similar to those in Figure 1 were constructed for the |^2^Π⟩ state of OH in field strengths over the range 0.0–0.2B_0_ with the internuclear axis aligned parallel to the field. Individual states were tracked along the potential energy curve using the maximum overlap method, generalized for use with complex orbitals.^16,109−112^ These are shown at field strengths of 0.1B_0_ and 0.2B_0_ in Figures 2 and 3, respectively.

#### 7.2.2 Dissociation Limit

For the atomic dissociation limit the energy is independent of the orientation with respect to the magnetic field and the energy of different atomic configurations can be easily calculated as a function of field strength. In eq 93 the dissociation limit of the |^2^Π⟩ state at zero field was identified as an O atom with *M*_*s*_ = −1 and the specific configuration O(2*p*_–1_^β^2*p*_0_^*αβ*^2*p*_+1_^β^). For single determinant models such as HF and CDFT this configuration has lower energy than, for example, the O(2*p*_–1_^*αβ*^2*p*_0_^β^2*p*_+1_^β^) and O(2*p*_–1_^β^2*p*_0_^β^2*p*_+1_^*αβ*^) configurations. This is a manifestation of the well-known multiplet problem for these methods—where the configurations contributing to the ^3^P state of the oxygen atom are not degenerate at zero field.^66,113^ By convention, quantities such as binding energies and atomization energies are calculated using the lowest energy configuration predicted by a given theory and this practice has been adopted in calculating the values of binding energy in Table 2.

In the presence of a magnetic field, not only is the degeneracy of multiplet components lifted but also each component may display a different variation in energy as a function of field. It is therefore interesting to consider the dissociation products with different multiplet components. For the |^2^Π⟩ state, the dissociation products given in eq 93 will have an initial variation in energy with field strength as
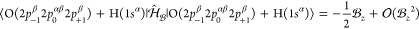
98In contrast, the dissociation products O(2*p*_–1_^*αβ*^2*p*_0_^β^2*p*_+1_^β^) + H(1*s*^α^) vary as
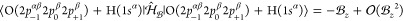
99We therefore expect that the energy of these dissociation products will fall below those in eq 93 as the magnetic field strength increases. As a result, care needs to be taken to select the correct atomic configurations in calculating quantities such as binding energies and atomization energies as a function of magnetic field.

**Figure 4:**
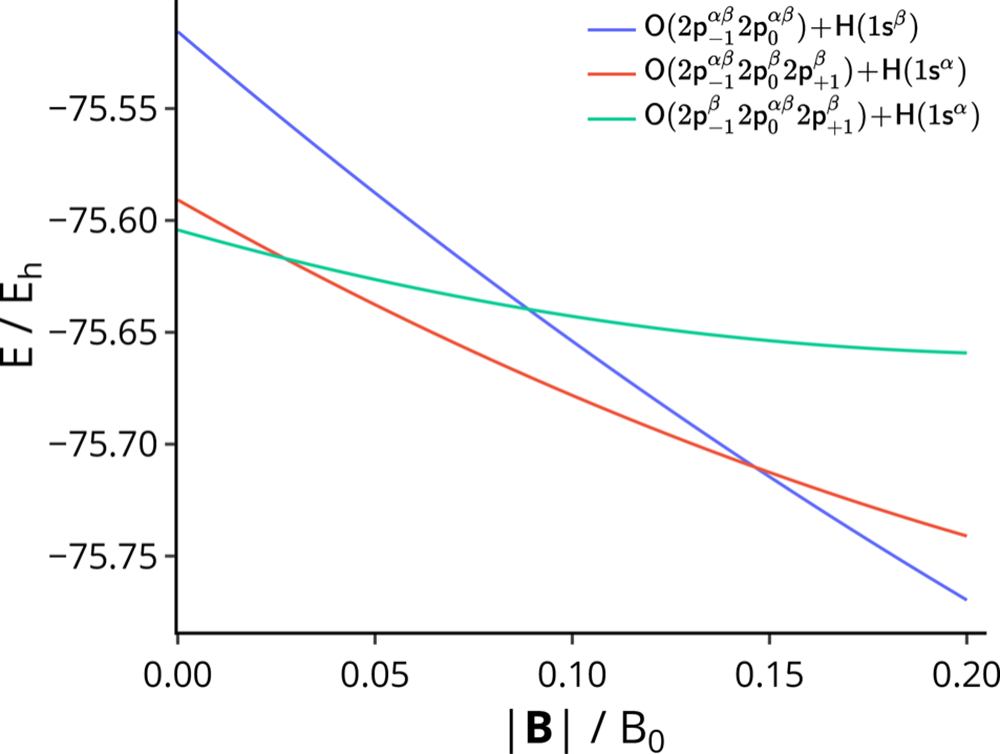
Sum of energies of the isolated atoms O and H in three possible configurations at dissociation as a function of field strength, computed with cTPSS.

The energy of the dissociation products of OH containing these different configurations of oxygen with *M*_*s*_ = −1 are shown as a function of field strength in Figure 4. It can be seen in Figure 4 that the change in the lowest-energy configuration of oxygen with *M*_*s*_ = −1 occurs at a field strength of 0.027B_0_ (compared to 0.008B_0_ with HF, shown in Figure S3). In the binding energies presented in Table 2, it is assumed that the dissociation products at each field strength contain the lowest-energy component of the multiplet; this is confirmed in Figures 2 and 3, which show that the |^2^Π⟩ state dissociates into the lower-energy *M*_*s*_ = −1 configuration of oxygen at those field strengths. We note that the multiplet problem for atomic species in magnetic fields has been previously observed by Ivanov and Schmelcher in, for example, refs (73) and (74).

In Figure 4 we also consider the dissociation products of the |^2^Σ⟩ state, which are much higher in energy than those of the |^2^Π⟩ state at zero field. However, they exhibit a more strongly paramagnetic behavior and the most rapid initial decrease in energy with magnetic field strength,
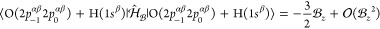
100which can be compared with eqs 98 and 99; since all three sets of dissociation products have *M*_*s*_ = −^1^/_2_, the differences in eqs 98–100 are due to the orbital paramagnetic interaction with the field. Accordingly, these dissociation products become the lowest in energy at a field strength of 0.146B_0_ (compared to 0.165B_0_ with HF, shown in Figure S3). This is reflected in Figure 3, which shows the potential energy curve of the |^2^Π⟩ state tending towards the higher of the two dissociation products. However, the equilibrium geometry of the ground state is correctly located in geometry optimization initialized from bond lengths of both 1.6 and 3.2 au, parallel to the field. This would suggest that the |^2^Π⟩ state is not crossed by another, tending to the lower-energy asymptote, at an internuclear distance of 3.2 au or less.

#### 7.2.3 OH in a Magnetic Field Perpendicular to the Bond

In a uniform magnetic field applied perpendicular to the internuclear axis of the OH molecule, a much greater reduction in symmetry occurs compared to the alignment of the field parallel to the internuclear axis. In this case, the *C*_∞_^ϕ^ axis is no longer a symmetry element. However, a mirror plane perpendicular to the magnetic field, in the plane of the internuclear axis does remain; the point group of the OH molecule with a magnetic field perpendicular to the internuclear axis thus becomes C_*s*_. This point group has only two irreducible representations, *A*′ and *A*″; the doubly degenerate Π irreducible representation of the zero-field C_∞*v*_ point group corresponds to a linear combination of the *A*′ and *A*″ irreducible representations in the C_*s*_ point group: Π → *A*′ + *A*″.

Furthermore, since the electrostatic potential and magnetic vector potential are no longer cylindrically symmetric around the internuclear axis, the orbital magnetic quantum number is not a good quantum number so the orbital paramagnetic contribution to the energy is less straightforward to evaluate. The reduced symmetry can allow a greater degree of mixing between orbitals to occur, in some cases resulting in an orbital angular momentum perpendicular to the internuclear axis through mixing with orbitals of higher angular momentum.^103,104^ The states themselves can be more difficult to identify as a result; however, analysis of the symmetry properties of the molecular orbitals of the two states that arise from the |^2^Π⟩ state upon application of a perpendicular magnetic field reveals their electronic configurations to be
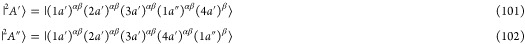
The |^2^*A*′⟩ state dissociates into O(2*p*_–1_^*αβ*^2*p*_0_^*αβ*^) + H(1*s*^β^), whereas the |^2^*A*″⟩ state dissociates into O(2*p*_–1_^*αβ*^ 2*p*_0_^β^2*p*_+1_^β^) + H(1*s*^α^). As shown in Figure 4 and discussed in Section 7.2.2, the first of these dissociation products drops in energy below the latter at field strengths of around 0.15–0.16B_0_.

In Figures 2 and 3, the potential energy curves are plotted for both the |^2^*A*′⟩ and |^2^*A*″⟩ states of OH in magnetic fields of strengths 0.1B_0_ and 0.2B_0_, respectively, oriented perpendicular to the internuclear axis. In addition, the equilibrium geometry obtained by geometry optimization from initial bond lengths of 1.6 and 3.2 au perpendicular to the field are plotted. It can be seen in both Figures 2 and 3 that the energy of the molecule at equilibrium is lower when aligned parallel to the field than perpendicular; however, the energy of the perpendicular orientation is stationary with respect to rotation relative to the field. The symmetry in this orientation is comparatively high since, upon rotation relative to the field, the system would lose its symmetry with respect to the plane perpendicular to the field and would be reduced to the C_1_ point group. Therefore, the geometry may be optimized perpendicular to the field if the initial geometry has this orientation.

Examination of the potential energy curves helps to the interpret the results of geometry optimizations in a perpendicular field. For the potential energy curve computed at 0.1B_0_, shown in Figure 2, the energy of the |^2^*A*′⟩ state in the perpendicular orientation is lowest at equilibrium but crosses the |^2^*A*″⟩ state at a bond length of around 3.31 au. Therefore, geometry optimization with an initial bond length of 3.2 au tracks the |^2^*A*′⟩ state and correctly identifies its equilibrium geometry, as is the case with an initial bond length of 1.6 au.

In a magnetic field of 0.2B_0_ perpendicular to the internuclear axis, the dissociation products of |^2^*A*′⟩ are lower in energy than those of |^2^*A*″⟩; however, the ordering of energies of the states at equilibrium is the same as that at 0.1B_0_. Therefore, there is no crossing of these two states along the potential energy curve as there is at 0.1B_0_. The potential energy curves for OH in a magnetic field of 0.2B_0_ perpendicular to the internuclear axis are shown in Figure 3; the equilibrium geometry of the |^2^*A*′⟩ state is correctly located from initial bond lengths of both 1.6 and 3.2 au in these conditions.

This simple example reveals some of the complexity associated with performing geometry optimization in a field. In a field, it is important not only that the initial geometry is sufficiently close to a local minimum for rapid convergence but also that the orientation of the magnetic field relative to the molecular frame is appropriate. Here we considered the high-symmetry parallel (ground-state) and perpendicular (excited-state) orientations. In general, it is necessary in the presence of a field to consider different starting orientations relative to the field to find the ground-state geometry. Furthermore, the resulting solutions should be carefully analyzed—for example in the perpendicular orientation at 0.1B_0_ the |^2^*A*′⟩ and |^2^*A*″⟩ states have similar energies at equilibrium and cross as the bond is stretched, so analysis is essential to ascertain which state is obtained in the geometry optimization. To facilitate this assignment, the consideration of the symmetry of the system in the presence of a magnetic field is invaluable. Despite this complexity the geometry optimization using analytic derivatives is able to efficiently locate all of the expected minima, confirming its utility for studying molecular structure and bonding in strong magnetic fields.

### 7.3 Ground-State Structure of Benzene

**Figure 5:**
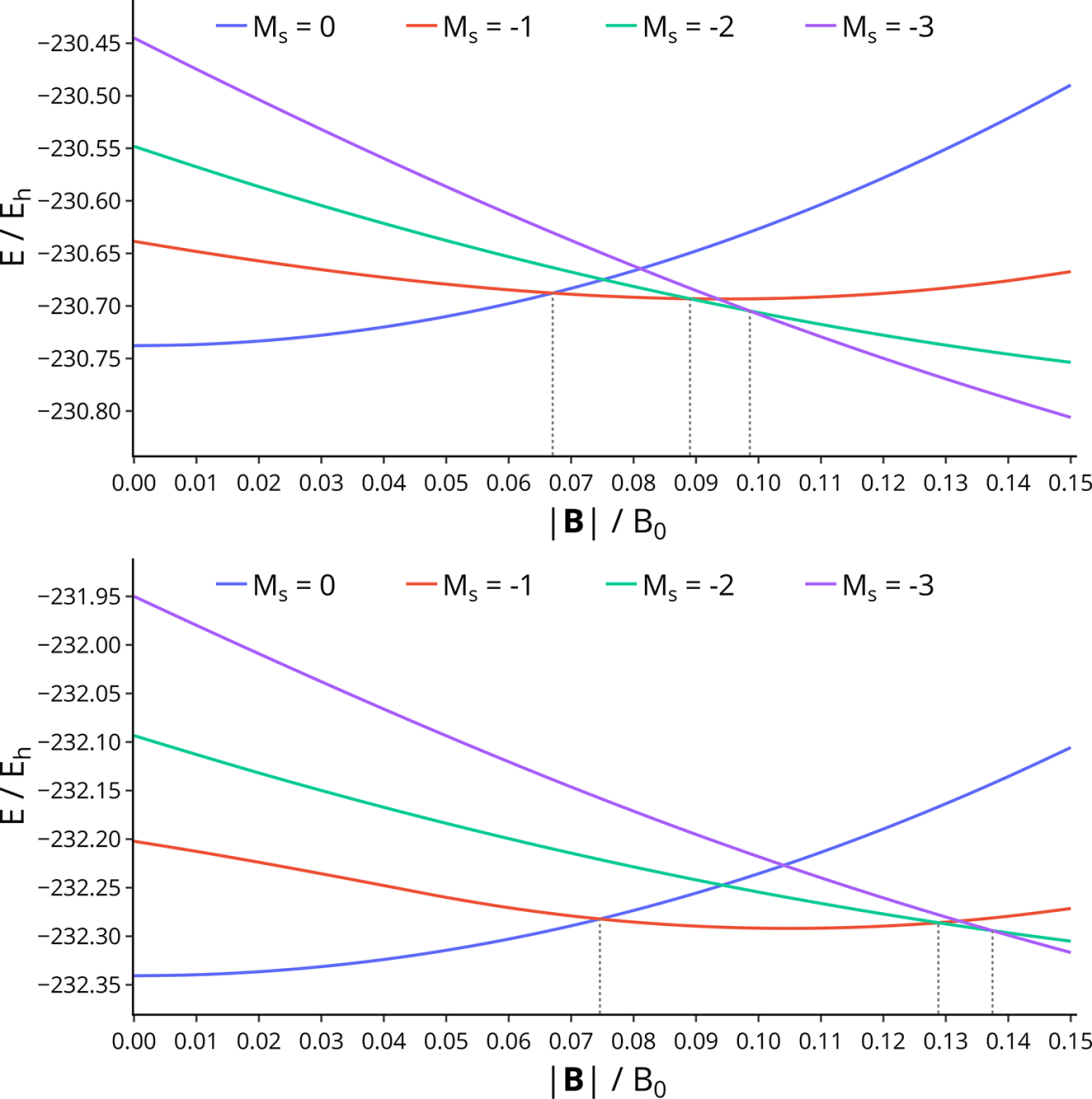
Energy as a function of field strength for the optimized geometries of benzene with *M*_*s*_ = 0, −1, −2, and −3, computed with Hartree–Fock (upper panel) and cTPSS (lower panel). The field strengths at which the ground-state spin projection changes are indicated with dashed lines between the energy at which this occurs and the horizontal axis.

In 2010 Caputo and Lazzeretti^114^ studied the geometrical effects of a magnetic field on the benzene molecule by considering the Lorentz force exerted on the atomic nuclei by the currents induced by the magnetic field. Tellgren et al.^3^ confirmed that the *M*_*s*_ = 0 state (the zero-field ground state) exhibits a shortening of the C–C bonds and extension of the C–H bonds in the presence of a magnetic field of 0.1B_0_ perpendicular to the molecular plane. Using the present implementation, which allows for unrestricted HF and CDFT optimizations, we investigate the behavior of not only the *M*_*s*_ = 0 state but also the *M*_*s*_ = −1, −2, and −3 states, in which two, four, and six of the π electrons are unpaired, respectively, as a function of magnetic field strength. In each case, the energy as a function of field strength is plotted for the optimized geometries in Figure 5.

As expected, the closed shell *M*_*s*_ = 0 state has an energy that rises diamagnetically. Consideration of the *M*_*s*_ = −1, −2, and −3 states highlights the importance of the spin–Zeeman effect in driving progressive unpairing of the π-electrons with increasing field strengths. For HF, the *M*_*s*_ = −1 state decreases in energy with field strength and becomes the ground state at 0.067B_0_. For states of higher spin projection, the decrease in energy with field strength is greater due to a larger spin–Zeeman effect; the *M*_*s*_ = −2 state becomes the ground state at 0.089B_0_, and the *M*_*s*_ = −3 state becomes the ground state at 0.099B_0_. All of the states considered become the ground state at |**B**| < 0.1B_0_.

While HF gives a qualitative description of the behavior of these states as a function of field, accounting for correlation has a significant effect on the quantitative picture; this can be seen by comparing the upper and lower panels of Figure 5. While qualitatively similar, the inclusion of correlation at the cTPSS level leads to substantial differences in the field strengths at which each *M*_*s*_ state becomes the ground state. In particular, the *M*_*s*_ = −1 state is the ground state over a much wider range of field strengths compared with that predicted by HF; this is principally due to the greater stabilization of the *M*_*s*_ = −1 state for cTPSS relative to HF. The *M*_*s*_ = −1 state becomes the ground state at 0.075B_0_, while the *M*_*s*_ = −2 state becomes the ground state at 0.129B_0_ and the *M*_*s*_ = −3 state becomes the ground state at 0.137B_0_.

**Figure 6:**
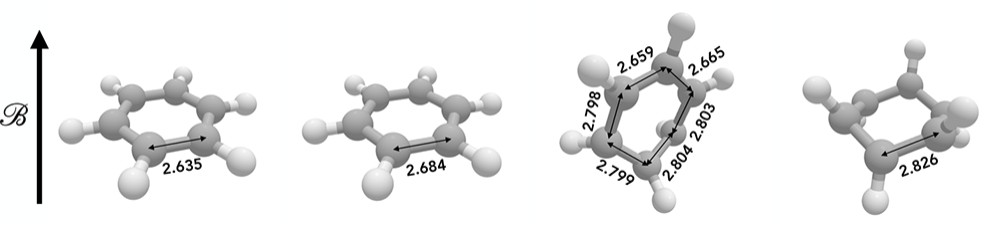
Optimized geometries of benzene in the presence of a 0.1 B_0_ magnetic field, computed with cTPSS, with *M*_*s*_ = 0, *M*_*s*_ = −1, *M*_*s*_ = −2, and *M*_*s*_ = −3, from left to right, respectively. Unique C–C bond lengths are indicated in au.

In Figure 6 we show the characteristic structures obtained for |**B**| = 0.1B_0_ for each *M*_*s*_ state. The structures obtained at the cTPSS level are qualitatively similar to those obtained at the HF level (see Figure S4 in the Supporting Information). Geometry optimization in a field determines not only the structural parameters of the molecule, such as bond lengths and angles, but also the preferred orientation of the molecule relative to the external magnetic field.

The *M*_*s*_ = 0 state has the familiar regular hexagonal arrangement of carbon atoms, with the plane of the molecule oriented perpendicular to the field. The point group of the nuclear framework is D_6*h*_, while the point group of the electronic structure in a magnetic field perpendicular to the plane of the molecule is C_6*h*_. For the *M*_*s*_ = −1 state, the π-system is disrupted by uncoupling two electrons, and as a result there are two unique C–C bond lengths: two of the C–C bonds (in the 1,4 configuration) are longer than the other four, forming an irregular hexagon. The zero-field molecular point group of this structure is D_2*h*_, which is reduced to C_2*h*_ in the magnetic field, to which it remains energetically favorable for the molecule to be oriented perpendicular.

The *M*_*s*_ = −2 state exhibits further disruption of the π-system since four electrons have been unpaired. The zero-field point group is C_*s*_, and the structure may be characterized as a half-chair structure. In contrast to the other spin projections, the molecule in the *M*_*s*_ = −2 state is oriented with the surface area perpendicular to the magnetic field minimized. As the field strength is increased, the orientation evolves such that the mirror plane in the molecular structure is increasingly parallel to the magnetic field. However, since this alignment does not become exact in the range of fields considered here, the overall point group of the structure is reduced to C_1_ in a magnetic field.

In the *M*_*s*_ = −3 state, all six of the π-electrons have been unpaired and there is no longer a π-system present; the optimized structure adopts a chairlike conformation. At zero field the point group of this structure would be D_3*d*_. In the presence of a magnetic field, the molecule is oriented such that its surface area perpendicular to the field is maximized; the principal axis returns to alignment with the field, with the result that the overall point group of the molecule in the field is S_6_.

**Figure 7:**
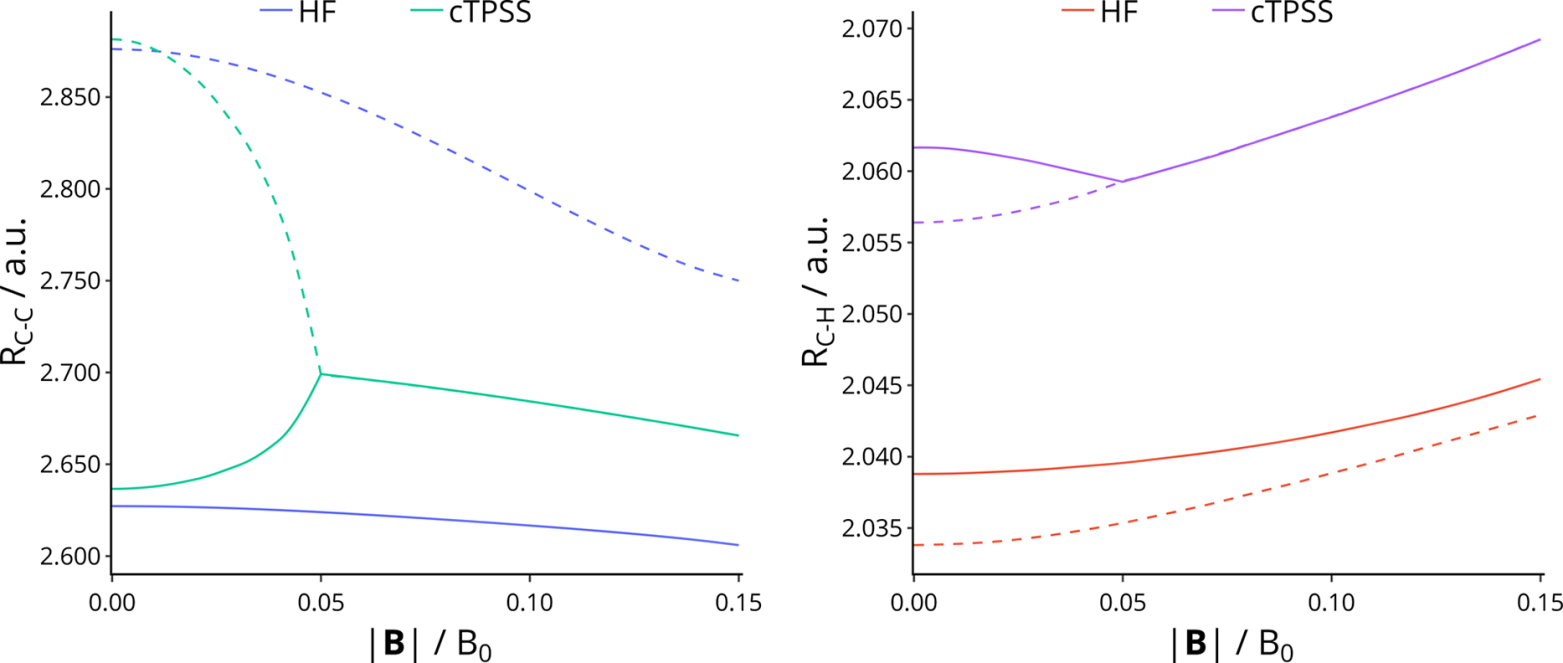
Equilibrium C–C (left) and C–H (right) bond lengths in benzene with *M*_*s*_ = −1 as a function of field strength perpendicular to the plane of the molecule (shown in Figures S4 and 6). In these structures, there are two unique C–C and C–H bond lengths; the solid lines indicate the length of four of the C–C or C–H bonds, while the dashed lines indicate the length of the other two C–C or C–H bonds.

While the HF and cTPSS structures are qualitatively similar for each *M*_*s*_, there are significant differences in quantitative values of the structural parameters such as the C–C and C–H bond lengths. Of particular interest is the variation of the optimized C–C and C–H bond lengths in the *M*_*s*_ = −1 state of benzene with magnetic field strength, shown in Figure 7. There are two sets of lines on each graph since there are two unique C–C and C–H bond lengths in the irregular hexagonal geometry of benzene; the dashed lines represent those for which there are two bonds of that length, whereas the solid lines represent those for which there are four bonds of that length. For this *M*_*s*_ state, the variation of the C–C and C–H bond lengths is significantly different for HF and cTPSS over the range of fields considered. In particular, in Figure 7 it can be seen that the cTPSS structure transitions from an irregular hexagon below ∼0.05 *B*_0_ to a regular hexagon at higher field strengths.

For the states with *M*_*s*_ = 0, −2, and −3 the variations of the C–C and C–H bond lengths are qualitatively similar at the HF and cTPSS levels. For the *M*_*s*_ = 0 state of benzene, the HF bond lengths are predicted to be consistently shorter than those from cTPSS. However, the variations of the C–C and C–H bond lengths with increasing field strength follow a similar trend in both cases, with C–C bond lengths decreasing and C–H bond lengths increasing; this is consistent with the behavior observed by Caputo and Lazzeretti^114^ and Tellgren et al.^3^ These are shown in Figure S5 of the Supporting Information.

For the *M*_*s*_ = −2 state, the low-symmetry C_1_ structure and rotation of the structure with changing field strength means that there is little further information that can be obtained from this analysis. For the *M*_*s*_ = −3 state, however, the higher-symmetry S_6_ structure has all equivalent C–C bonds and all equivalent C–H bonds. The C–H bond lengths decrease monotonically with increasing magnetic field strength; the HF bond lengths are consistently shorter than those from cTPSS. The C–C bond lengths show a more complex behavior, first decreasing with field strength before beginning to increase at higher fields, with the HF bond lengths consistently longer than those from cTPSS; this can be seen in Figure S6 of the Supporting Information. The complex behavior of the bonding in this state is interesting and the development of tools for analysis of chemical bonding in strong magnetic fields a focus for future work.

## 8 Conclusions

In this work, a simple and computationally tractable approach for the evaluation of derivative integrals over LAOs, used in electronic structure calculations in the presence of strong magnetic fields, has been developed and implemented. This builds on previous work in developing an efficient framework for the evaluation of the molecular integrals themselves over LAOs presented in ref (15). The principal focus here is on the generalized McMurchie–Davidson algorithm^1,33,34,37−40,115,116^ due to its amenability to the evaluation of derivative integrals; however, due to the superior efficiency of the Rys quadrature^30,51−53,117^ for integrals of high angular momentum, a method using a hybrid of Rys and McMurchie–Davidson approaches was proposed for the evaluation of derivative integrals over LAOs.

The geometrical gradient of the electronic energy for Hartree–Fock in the presence of a strong magnetic field using LAOs^3^ was implemented in this work, both in the conventional form using the derivatives of four-index ERIs but also in the RI approximation, generalizing the principle of approximating ERIs over LAOs using the RI approximation^27,30^ to the construction of the full analytical gradient using two- and three-center derivative integrals. In addition, full analytical first derivatives of the exchange–correlation energy in CDFT were presented for the first time, available for functionals of the LDA, GGA, and meta-GGA types and their (range-separated) hybrids including current-dependent contributions. This CDFT implementation constitutes a cost-effective family of methods for studying molecular structure in strong magnetic fields.

To provide an illustration of the interesting chemical phenomena that these developments allow the exploration of, a detailed analysis was presented for the optimized geometries of two molecules in a range of magnetic field strengths. In the first of these, the OH diatomic molecule, it was shown how the magnetic field can profoundly affect the electronic state and bonding in the molecule. For the atomic dissociation limit the ground-state energies are independent of orientation relative to the field; however, as the atoms become bound, the system gains a preferred orientation relative to the field. This additional effect, in combination with the usual Coulombic bonding interactions, leads to a rather complex chemistry. Local stationary points were located for several low-lying states with a magnetic field aligned parallel and perpendicular to the internuclear axis. Calculating the underlying potential energy curves explicitly confirmed the accuracy of the geometry optimization using analytical gradients. In addition, this analysis highlighted the importance of the initial guess geometry and orientation relative to the field in these calculations. In many regards, the challenges for optimizing structures in the presence of magnetic fields are akin to those found in excited-state geometry optimization, requiring good initial guesses for the structures in the vicinity of desired stationary points and careful analysis to identify the electronic states obtained following the optimization. The reduction in symmetry that can occur upon application of a magnetic field further complicates matters. Nonetheless, the implementation in the present work offers the possibility to rapidly locate the relevant stationary points, which can be readily characterized by their symmetry properties, opening-up the possibility of exploring the exotic but rich chemistry of systems in the presence of strong magnetic fields.

In the second example, the geometry of benzene was optimized at a range of field strengths for states with several spin projections. It was shown that the ground-state spin projection and structure evolve from the familiar hexagonal structure at low field with *M*_*s*_ = 0, through a distorted hexagon with *M*_*s*_ = −1, to a half-chair conformation with *M*_*s*_ = −2 at intermediate fields, before adopting a chairlike structure with *M*_*s*_ = −3 at higher fields. These structures reflect the disruption of the π-system as it becomes more and more favorable to unpair electrons in stronger fields. While HF and cTPSS calculations revealed a similar qualitative picture, their comparison showed that the inclusion of correlation can have a significant effect on the predictions of the field strengths at which each state becomes the ground state. A detailed analysis of the bonding in each structure was presented, extending over previous analysis in the literature for the *M*_*s*_ = 0 state.^3,114^

Access to the efficient evaluation of molecular gradients in the presence of strong magnetic fields enables a wide range of applications to be considered. These include the study of chemical reactivity by searching for minima, transition states, and intrinsic reaction coordinates in strong fields, excited-state geometry optimization in strong fields, *ab initio* molecular dynamics in strong fields, and coupling of these approaches to real-time electronic structure methods under these conditions. Enabled by the developments presented here, these topics are the focus of ongoing investigation, the results of which will be presented in future work.

## Supporting Information Available

SI The Supporting Information is available free of charge at https://pubs.acs.org/doi/10.1021/acs.jctc.0c01297.

Potential energy curves and optimized geometries of OH in 0.1B_0_ and 0.2B_0_ magnetic fields parallel and perpendicular to the bond, computed with Hartree−Fock; plot of the energies of OH dissociation products with varying field strength computed with Hartree−Fock; optimized structures of benzene with *M*_s_ = 0, −1, −2, and −3 in a 0.1B_0_ magnetic field computed with Hartree−Fock; plots of optimized C−C and C−H bond lengths in benzene with *M*_s_ = 0 and −3 with varying field strength (PDF)
