# Supplementary material for: Optimizing Molecular Geometries in Strong Magnetic Fields
Source: J Chem Theory Comput. 2021 Mar 16;17(4):2166–85. doi: 10.1021/acs.jctc.0c01297 (PMC8047810; doi:10.1021/acs.jctc.0c01297)
Supplement: Supplementary file 1 — ct0c01297_si_001.pdf [file ct0c01297_si_001.pdf]

Optimizing Molecular Geometries In Strong Magnetic Fields

Tom J. P. Irons,*,† Grégoire David,† and Andrew M. Teale*,†,‡

†*School of Chemistry, University of Nottingham, University Park, Nottingham, NG7 2RD, United Kingdom*

‡*Hylleraas Centre for Quantum Molecular Sciences, Department of Chemistry, University of Oslo, P.O. Box 1033 Blindern, N-0315 Oslo, Norway*

E-mail: tom.irons@nottingham.ac.uk; andrew.teale@nottingham.ac.uk

S1 Equilibrium Geometry of OH

For the potential energy curves computed with HF at 0.1 B_0, shown in Figure S1, the energy of the $|^2A'\rangle$ state in the perpendicular orientation is lowest at equilibrium but crosses the $|^2A''\rangle$ state at a bond length of around 2.62 au. Therefore, the geometry optimization from an initial O-H bond length of 3.2 au tracks the $|^2A''\rangle$ state, which is the lowest in energy at this point. However, since the potential energy curve of this state has a local maximum at around 2.97 au, the geometry optimization cannot locate the equilibrium geometry of this state and instead tracks its potential energy curve towards dissociation. This is in contrast to the picture obtained cTPSS, with which geometry optimization correctly locates the equilibrium geometry from both starting points.

The potential energy curves computed with HF, shown in Figure S2 suggest that $|^2A'\rangle$ is lowest in energy across the entire potential energy curve with a perpendicular field of 0.2 B_0. However, it can be seen that geometry optimization from an initial bond length of 3.2

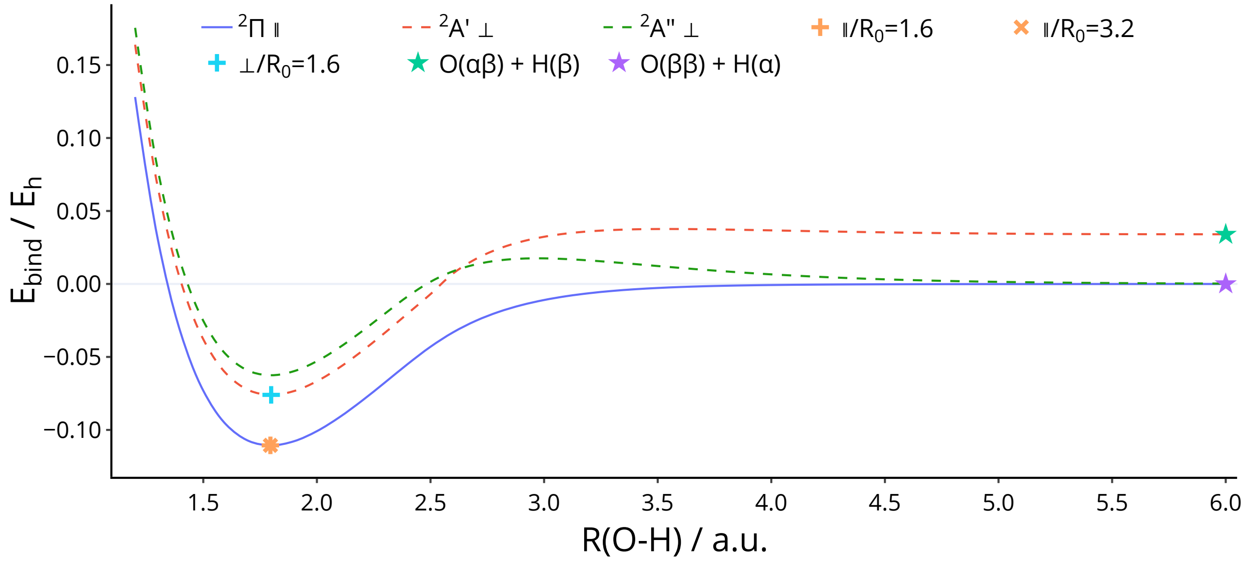

Figure S1: Potential energy curve of OH in a field of 0.10 B_0 parallel and perpendicular to the O-H axis, computed with HF. Symbols $+$ and $\times$ represent the equilibrium geometries obtained by geometry optimization from initial bond lengths of 1.6 and 3.2 au, respectively. The superposition of both solutions appears as an $*$ symbol. $\mathrm{O}(2p_{-1}^{\alpha\beta}2p_0^{\beta}2p_{+1}^{\beta}) + \mathrm{H}(1s^{\alpha})$ and $\mathrm{O}(2p_{-1}^{\alpha\beta}2p_0^{\alpha\beta}) + \mathrm{H}(1s^{\beta})$ are abbreviated as $\mathrm{O}(\beta\beta) + \mathrm{H}(\alpha)$ and $\mathrm{O}(\alpha\beta) + \mathrm{H}(\beta)$, respectively.

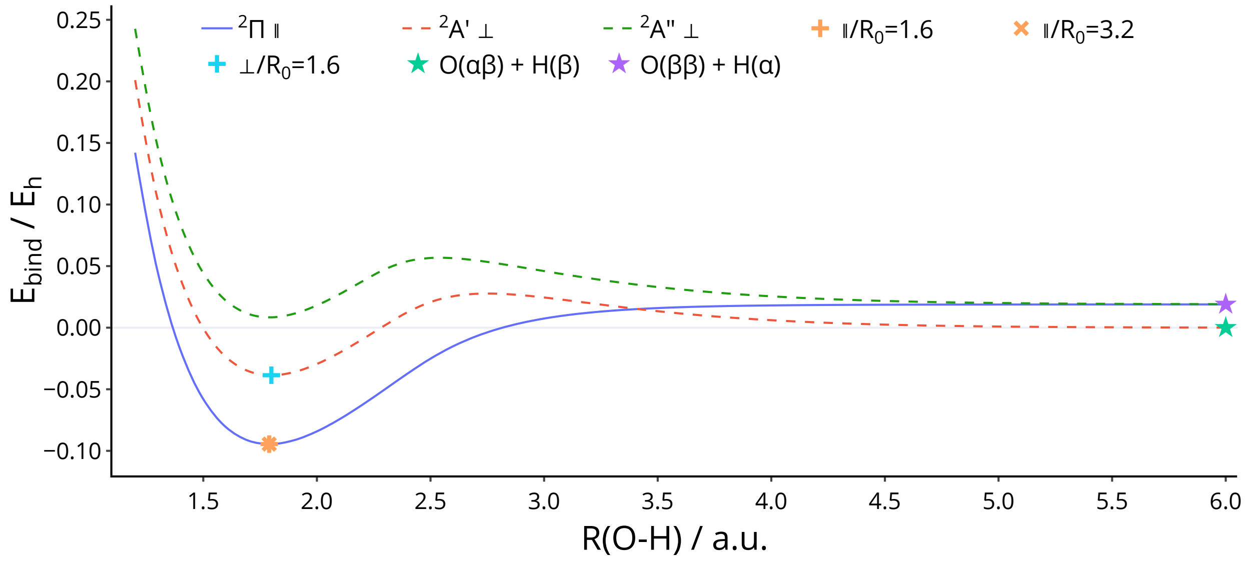

Figure S2: Potential energy curve of OH in a field of 0.20 B_0 parallel and perpendicular to the O-H axis, computed with HF. Symbols $+$ and $\times$ represent the equilibrium geometries obtained by geometry optimization from initial bond lengths of 1.6 and 3.2 au, respectively. The superposition of both solutions appears as an $*$ symbol. $\mathrm{O}(2p_{-1}^{\alpha\beta}2p_0^{\beta}2p_{+1}^{\beta}) + \mathrm{H}(1s^{\alpha})$ and $\mathrm{O}(2p_{-1}^{\alpha\beta}2p_0^{\alpha\beta}) + \mathrm{H}(1s^{\beta})$ are abbreviated as $\mathrm{O}(\beta\beta) + \mathrm{H}(\alpha)$ and $\mathrm{O}(\alpha\beta) + \mathrm{H}(\beta)$, respectively.

au with a perpendicular field of 0.2 B_0 does not locate the equilibrium geometry of the $|^2A'\rangle$ state. This is not due to a crossing between the states; instead it is because there is a local maximum on the potential energy curve of this state at around 2.75 au so the geometry optimization from an initial bond length of 3.2 au tracks the descent in energy towards the dissociation limit. The presence of this feature in the potential energy curve of OH is likely due to the underbinding of the molecular state by HF relative to the dissociation products; using cTPSS, the equilibrium geometry is correctly located from both starting points.

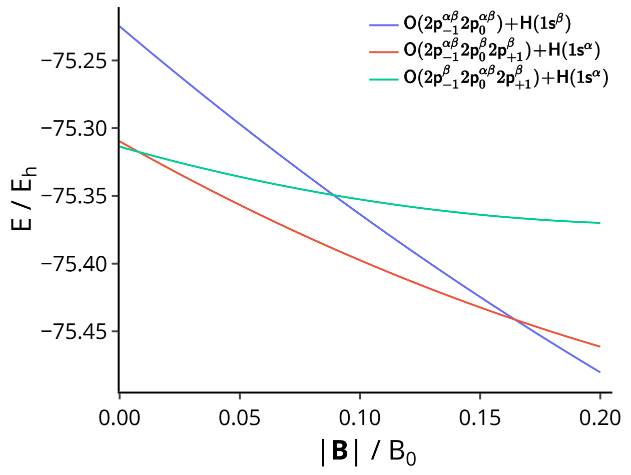

Figure S3: Sum of energies of the isolated atoms O and H in three possible configurations at dissociation as a function of field strength, computed with HF.

S2 Ground State Structure of Benzene

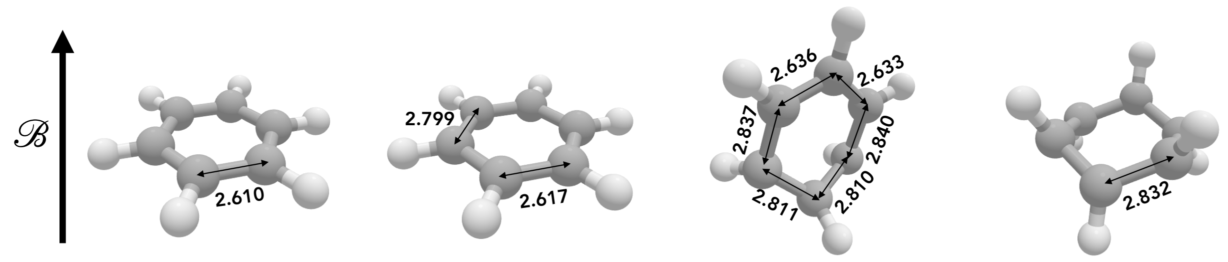

Figure S4: Optimized geometries of benzene in the presence of a 0.1 B_0 magnetic field, computed with Hartree-Fock, with $M_s = 0$, $M_s = -1$, $M_s = -2$ and $M_s = -3$, from left to right respectively. Unique C-C bond lengths are indicated in au.

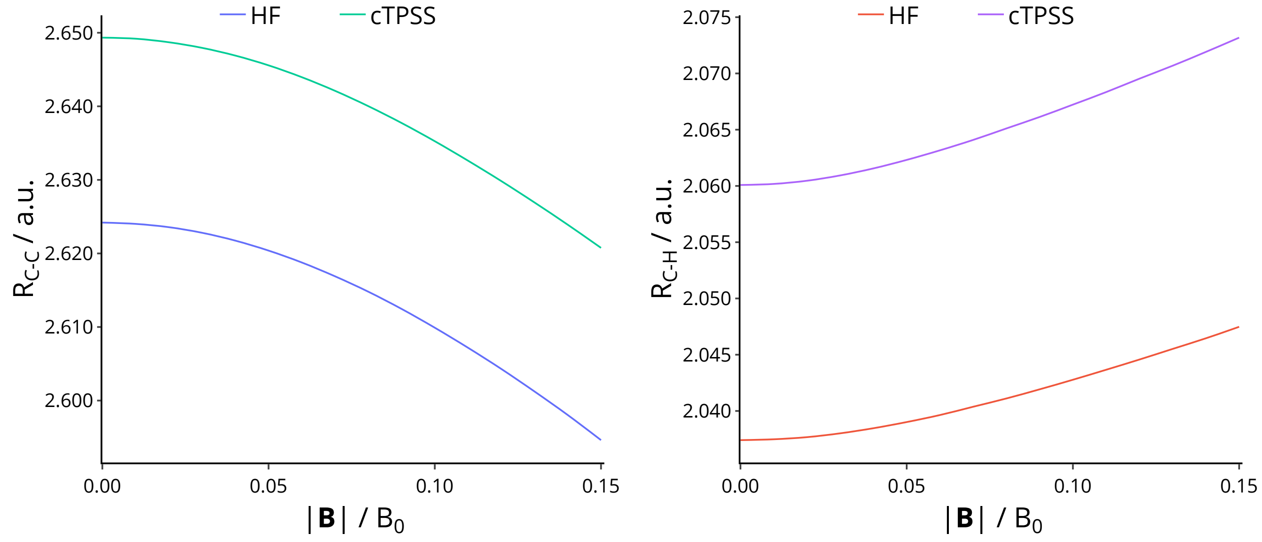

Figure S5: Equilibrium C-C (left) and C-H (right) bond lengths in benzene with $M_s = 0$ as a function of field strength perpendicular to the plane of the molecule (shown in Figures S4 and 6).

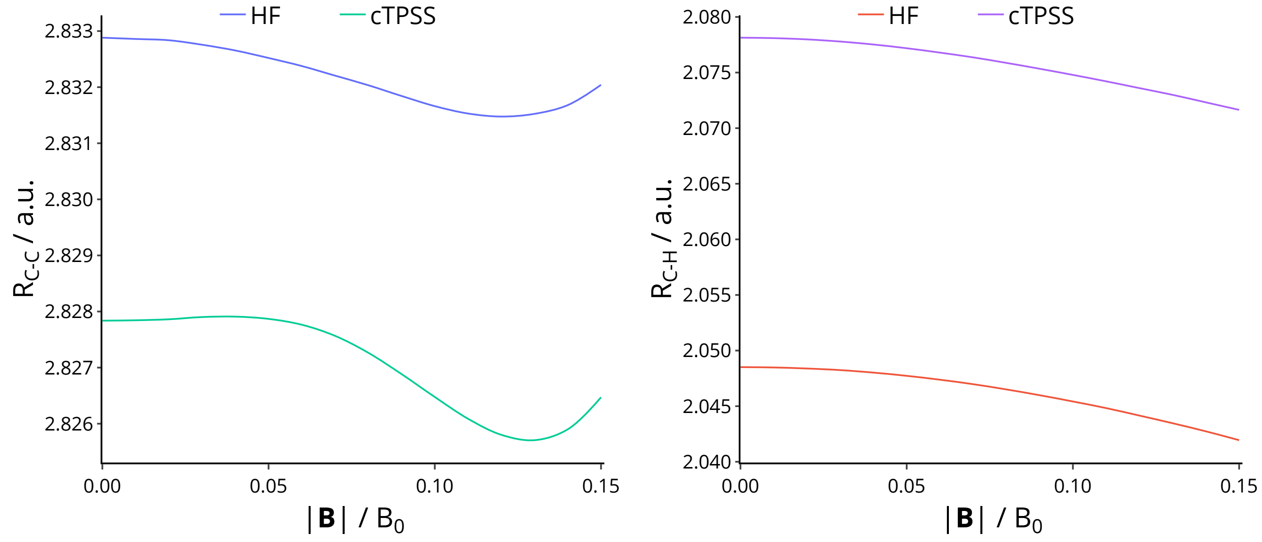

Figure S6: Equilibrium C-C (left) and C-H (right) bond lengths in benzene with $M_s = -3$ as a function of field strength (with orientation shown in Figures S4 and 6).
